# Defect Detection in GIS X-Ray Images Based on Improved YOLOv10

**DOI:** 10.3390/s25175310

**Published:** 2025-08-26

**Authors:** Guoliang Xu, Xiaolong Bai, Menghao Huang

**Affiliations:** School of Communications and Information Engineering, Chongqing University of Posts and Telecommunications, Chongqing 400065, China; s230101002@stu.cqupt.edu.cn (X.B.); s230132048@stu.cqupt.edu.cn (M.H.)

**Keywords:** defect detection, Gas-Insulated Switchgear (GIS), YOLOv10, MCAttn, PPA

## Abstract

Timely and accurate detection of internal defects in Gas-Insulated Switchgear (GIS) with X-ray imaging is critical for power system reliability. However, automated detection faces significant challenges from small, low-contrast defects and complex background structures. This paper proposes an enhanced object-detection model based on the lightweight YOLOv10n framework, specifically optimized for this task. Key improvements include adopting the Normalized Wasserstein Distance (NWD) loss function for small object localization, integrating Monte Carlo (MCAttn) and Parallelized Patch-Aware (PPA) attention to enhance feature extraction, and designing a GFPN-inspired neck for improved multi-scale feature fusion. The model was rigorously evaluated on a custom GIS X-ray dataset. The final model achieved a mean Average Precision (mAP) of 0.674 (IoU 0.5:0.95), representing a 5.0 percentage point improvement over the YOLOv10n baseline and surpassing other comparative models. Qualitative results also confirmed the model’s enhanced capability in detecting challenging small and low-contrast defects. This study presents an effective approach for automated GIS defect detection, with significant potential to enhance power grid maintenance efficiency and safety.

## 1. Introduction

Gas-Insulated Switchgear (GIS), characterized by its compact design, high integration level, stable performance, and low maintenance costs, has been widely adopted in modern power systems [1]. As critical nodes in the power grid, the safe and stable operation of GIS directly impacts the reliability of the entire power system. However, during manufacturing, transportation, installation, and long-term operation, internal defects such as metallic particles, sharp protrusions on conductors, bubbles or cracks within insulating components, and foreign objects can arise in GIS [1,2]. If not detected promptly, these potential defects can escalate into severe faults, jeopardizing grid security. Therefore, regular and effective internal condition monitoring of GIS, particularly the early identification and diagnosis of defects, is paramount for implementing preventive maintenance and ensuring power grid safety.

Multiple diagnostic methods are currently available for assessing the internal state of Gas-Insulated Switchgear (GIS), including Ultra-High Frequency (UHF) detection of partial discharges (PD) detection, ultrasonic detection, and $SF_{6}$ gas decomposition product detection [2]. While these methods are effective in detecting discharge-related insulation defects, their capability is limited for physical defects that do not produce significant discharge signals, such as minor structural deformations, non-discharging foreign objects, or incipient cracks. In contrast, X-ray digital imaging modalities, such as Computed Radiography (CR) and Digital Radiography (DR), serve as non-contact, non-destructive inspection tools capable of penetrating the GIS metallic enclosure to directly visualize various physical defects, regardless of whether they generate discharges [3]. This endows X-ray inspection with unique advantages and substantial application potential in the field of GIS defect diagnosis [2]. Nevertheless, automated defect recognition in X-ray images poses three major challenges. First, the minute size and morphological diversity of critical defects (e.g., gas voids, micro-cracks, or metallic particles) may result in representations spanning only a few pixels [4]. Second, the low contrast caused by minimal X-ray absorption differences between defects and surrounding materials leads to blurred feature boundaries. Finally, the intricate GIS internal architecture introduces overlapping projections and complex background noise, which may occlude or mimic defect signatures. The interpretation of X-ray images currently relies heavily on manual expertise, which is inefficient, costly, and subjective [2], urgently necessitating the development of automated and intelligent detection technologies.

Traditional image processing-based defect-detection algorithms, such as threshold segmentation, morphological filtering, and edge detection [5,6], while applicable in certain specific scenarios, are generally highly sensitive to image quality and parameter settings. Other recent approaches have also explored novel paradigms such as training-free learning for GIS image analysis [7]. However, these methods can be inadequate for handling the full complexity and diversity of GIS X-ray images. In recent years, deep learning, particularly Convolutional Neural Networks (CNNs), has emerged as the mainstream approach in object detection [4]. Single-stage detectors, notably the You Only Look Once (YOLO) series [4], have garnered significant attention for their effective balance between detection speed and accuracy. From the initial YOLOv1 [8] and YOLOv3 [9], to YOLOv4 [10] and YOLOv5 [11], which incorporated CSPNet [12] and PANet [13], and more recently YOLOv8 [14], YOLOv9 [15], and YOLOv10 [16], and the very recent YOLOv11 [17], and even YOLOv12 [18], the YOLO framework has continuously evolved with steadily improving performance [4]. Previous research has applied YOLOv5 to pattern recognition of GIS partial discharge PRPD patterns [1], demonstrating the potential of the YOLO framework in this domain. However, directly applying general-purpose YOLO models to raw X-ray images for defect detection still encounters the aforementioned challenges: standard loss functions are insensitive to minuscule targets [19]; fixed network architectures may not optimally extract low-contrast, detail-rich X-ray features; and standard multi-scale fusion mechanisms (e.g., FPN [20], PANet [13]) may not be optimal for GIS defects, which exhibit significant size variations [21]. Therefore, in-depth optimization of YOLO models tailored to the characteristics of GIS X-ray images is essential.

To address the aforementioned issues and challenges, this paper adopts the lightweight and high-performance YOLOv10n as its baseline model. This choice was made based on the technological context at the time the experimental design and model development were finalized, when YOLOv10n provided an optimal trade-off between accuracy, speed, and computational efficiency for our specialized task. Although YOLOv11 [17] and YOLOv12 [18] have since been released, integrating them into this study would require a complete re-tuning of the training pipeline, hyperparameters, and improvement modules, which lies beyond the scope of this work. Our focus here is to demonstrate the effectiveness of the proposed improvement strategies on a stable and well-established baseline. Nevertheless, the extension and evaluation of these strategies on the latest YOLO architectures will be an important direction for future research. Building upon this baseline, we propose a series of synergistic improvement strategies aimed at constructing an enhanced model more suitable for defect detection in GIS X-ray images. The key enhancements comprise: adopting the Normalized Wasserstein Distance (NWD) loss function to strengthen the localization capability for small targets; introducing Monte Carlo Attention (MCAttn) [22] and Parallelized Patch-Aware Attention (PPA) [23] into the backbone network to enhance feature discriminability and robustness in complex backgrounds; and designing a novel neck network structure inspired by the concept of enhanced multi-scale interaction from the Generalized Feature Pyramid Network (GFPN) [21]. Through the organic integration of these modules, we aim to achieve high-precision, high-efficiency detection of various internal defects in GIS, especially for challenging samples.

The main contributions of this paper can be summarized as follows:

- A deeply improved object-detection model based on YOLOv10n is proposed, specifically tailored to the characteristics of GIS X-ray images.
- The NWD loss function is applied to the task of GIS X-ray defect detection, and its effectiveness in enhancing the localization accuracy of minute defects is validated.
- Two advanced attention mechanisms, MCAttn and PPA, are integrated into the backbone network of YOLOv10, exploring and demonstrating their potential in enhancing feature representation for X-ray images.
- A neck network structure inspired by the GFPN concept is designed and implemented to improve the fusion of multi-scale defect features.
- Comprehensive experimental validation is conducted on a GIS X-ray dataset collected from real-world industrial scenarios, with results indicating that the proposed model significantly outperforms the baseline and several other state-of-the-art models of similar types.

The remainder of this paper is organized as follows: Section 2 details the proposed model architecture and its key improved components. Section 3 describes the dataset used for experiments, evaluation metrics, and specific implementation details. It also provides an in-depth analysis of the experimental results, including ablation studies and performance comparisons with existing advanced methods. Section 4 summarizes the entire work and outlines future research directions.

## 2. Proposed Method

To address the challenges in detecting defects in GIS X-ray images, which are inherently characterized by small target sizes, low contrast, and complex backgrounds, this paper proposes an enhanced defect-detection method based on the YOLOv10n framework. To improve the model’s performance on this specific task, we have systematically enhanced the baseline model (YOLOv10n), focusing on three core aspects:

1. Loss Function: At the loss function level, the NWD loss is adopted to replace traditional bounding box losses based on Intersection over Union (IoU), to better address the localization issues of small targets.
2. Backbone Network: At the backbone network level, the MCAttn mechanism is incorporated into the core C2f feature extraction modules, replacing the original C2f modules at the 2nd, 4th, and 6th layers, and PPA is used to replace the C2f module at the 8th layer. This aims to enhance the network’s ability to extract and perceive critical defect features.
3. Neck Structure: At the neck network level, a new multi-scale feature fusion structure is designed, inspired by the principles of GFPN, to improve adaptability to defects of varying sizes.

**Figure 1:**
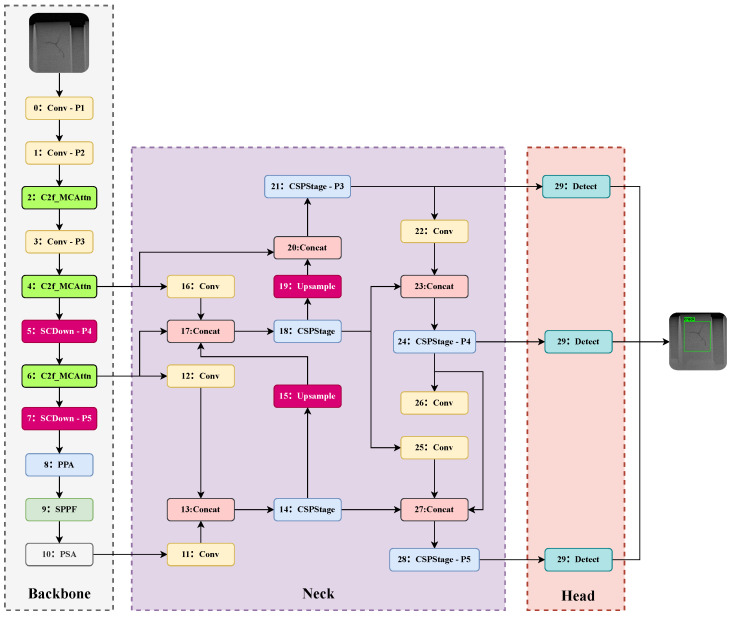
Improved YOLOv10 network model structure.

The overall network architecture of the proposed method is illustrated in Figure 1. This architecture integrates all the aforementioned improvements. The subsequent subsections will elaborate on the specific design of the NWD loss function application (Section 2.1), the attention mechanism enhancements in the backbone network (Section 2.2 and Section 2.3), and the GFPN-inspired neck network structure (Section 2.4).

### 2.1. NWD Loss

Common bounding box regression loss functions in object detection, such as those based on IoU such as GIoU, DIoU, and CIoU, perform well in general object-detection tasks. However, they exhibit certain limitations when dealing with small defect targets commonly found in GIS X-ray images, as in this study. Specifically, these loss functions are highly sensitive to the positional offsets of small targets; even minor pixel deviations can cause drastic changes in the IoU value, potentially reducing it to zero. This instability complicates model training and hinders the precise regression of small target bounding boxes.

To overcome this challenge, this paper introduces NWD [19] as both a metric and a loss function for bounding boxes. The core idea of NWD is to model a bounding box R=(cx,cy,w,h) (where (cx,cy) are the center coordinates, and *w* and *h* are the width and height, respectively) and its internal pixel weight distribution as a two-dimensional Gaussian distribution N(μ,Σ). The mean is $\mu ={\left[cx,cy\right]}^{T}$, and the covariance matrix is $\Sigma =\mathrm{diag}(\left[w^{2}/4,h^{2}/4\right])$. Instead of directly calculating the overlapping area, NWD employs the Wasserstein distance (also known as Earth Mover’s distance) to measure the similarity between the Gaussian distributions $N_{p}$ and $N_{gt}$ corresponding to two bounding boxes (e.g., a predicted box *p* and a ground truth box gt). The squared second-order Wasserstein distance between them, $W_{2}^{2}\left(N_{p},N_{gt}\right)$, can be efficiently computed as:

(1) $$W_{2}^{2}\left(N_{p},N_{gt}\right)=∥\mu_{p}-\mu_{gt}{∥}_{2}^{2}+{∥\frac{1}{2}{\left[w_{p},h_{p}\right]}^{T}-\frac{1}{2}{\left[w_{gt},h_{gt}\right]}^{T}∥}_{2}^{2}$$

where $\mu_{p}$ and $\mu_{gt}$ are the center coordinate vectors of the predicted box and the ground-truth box, respectively; $\left(w_{p},h_{p}\right)$ and $\left(w_{gt},h_{gt}\right)$ are their corresponding width and height; and ${∥\cdot ∥}_{2}^{2}$ denotes the squared Euclidean distance. This distance, as shown in Equation (1), considers both the deviation in center points and the differences in the dimensions of the bounding box (width and height).

Compared to IoU-based losses, NWD offers significant advantages, particularly in small object detection. (1) Even if two bounding boxes do not overlap at all (where the IoU gradient would be zero), NWD can still provide smooth and informative gradients, thereby facilitating continuous model optimization. (2) NWD is insensitive to object scale and provides a more principled way to measure the similarity between small targets, exhibiting greater robustness to positional and size deviations of minute objects. These characteristics make NWD highly suitable for enhancing the localization accuracy of small defects in GIS X-ray images.

Based on the Wasserstein distance, the NWD similarity is defined as:

(2) $$NWD\left(N_{p},N_{gt}\right)=\exp\left(-\frac{\sqrt{W_{2}^{2}\left(N_{p},N_{gt}\right)}}{C}\right)$$

where $N_{p}$ and $N_{gt}$ represent the Gaussian distributions corresponding to the predicted and ground-truth boxes, $W_{2}^{2}\left(\cdot ,\cdot \right)$ is the squared Wasserstein distance from Equation (1), and *C* is a constant used to scale the distance, typically set based on the statistics of the dataset (e.g., average target size). The similarity of the NWD ranges from (0,1], where a value closer to 1 indicates a greater similarity between the two boxes. Finally, the NWD loss function $L_{NWD}$ (where L denotes loss) adopted in this paper is calculated as follows:

(3) $$L_{NWD}=1-NWD\left(N_{p},N_{gt}\right)$$

where $L_{NWD}$ is the final NWD loss, and $NWD(N_{p},N_{gt})$ is the similarity metric defined in Equation (2). The range of this loss function $L_{NWD}$ is [0,1); a higher NWD similarity results in a smaller loss value. In the model proposed in this paper, we replace the original bounding box regression loss branch of YOLOv10n with this $L_{NWD}$ loss (see Equation (3)), aiming to achieve superior detection performance for minute defects.

### 2.2. C2f_MCAttn Module

To further enhance the model’s capability to extract subtle defect features from complex GIS X-ray backgrounds, this paper improves the core Convolutional-to-Feature (C2f) modules within the YOLOv10n backbone network. Although standard C2f modules achieve a balance between efficiency and performance, their feature extraction ability still has room for improvement when processing X-ray images with low signal-to-noise ratios and poor feature discriminability, often leading to the neglect of critical defect details or interference from background textures.

**Figure 2:**
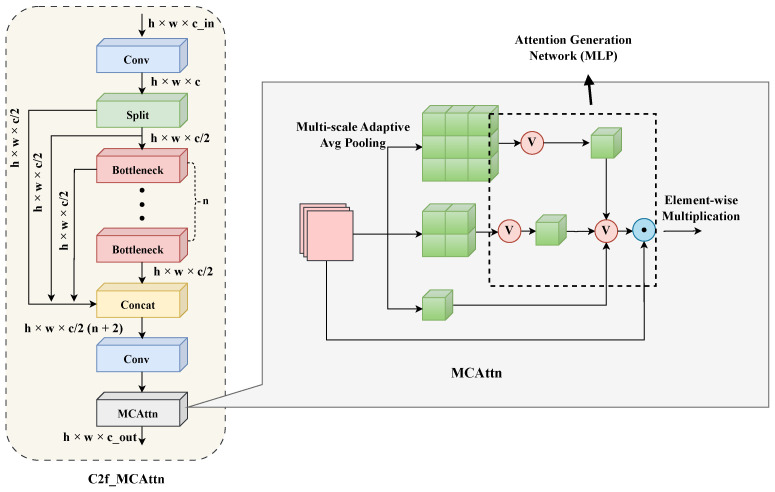
Structure of C2f_MCAttn module.

For this purpose, we chose to embed the MCAttn mechanism [22] within the C2f modules used at the P2, P3, and P4 feature levels of the backbone network (corresponding to the 2nd, 4th, and 6th layers in the overall architecture shown in Figure 1). This forms the C2f_MCAttn unit (its structure is shown on the left side of Figure 2). Unlike the baseline C2f, the MCAttn module is deployed after the final 1 × 1 convolutional layer of the C2f unit, performing dynamic, data-driven feature recalibration on the module’s output high-level features $X\in R^{C\times H\times W}$.

The core innovation of the MCAttn mechanism [22] lies in its use of randomization strategies and multi-scale information aggregation to generate attention weights that are more robust and insensitive to scale variations. Its key operational principles can be broken down into the following steps, as illustrated in Figure 2:

1. Multi-Scale Contextual Feature Extraction: As depicted in the right panel of Figure 2, the process begins with multi-scale feature extraction. The input features X are passed through parallel adaptive average pooling operations with different output spatial resolutions (e.g., a preset set S={1×1,2×2,3×3}). This step, represented by the “Pooling” block in the diagram, captures feature statistical summaries $\left\{{\mathrm{Pool}}_{s}\left(X\right)|s\in S\right\}$ under different receptive fields.
2. Monte Carlo Attention Sampling: During the model training phase, one or a combination of feature representations $z_{sampled}$ is randomly sampled from the aforementioned set of multi-scale feature summaries. This stochastic sampling enhances the model’s robustness and prevents overfitting to specific patterns in the training data.
3. Channel Attention Generation and Application: The sampled features $z_{sampled}$ are then fed into a lightweight attention generation network. This network, represented by the subsequent series of operations in the diagram, is typically a Multi-Layer Perceptron (MLP) inspired by the Squeeze-and-Excitation (SE) [24] structure. It comprises two linear layers and non-linear activations to learn the importance of each channel, ultimately outputting a channel attention vector $w=\sigma \left(\mathrm{MLP}\left(z_{sampled}\right)\right)\in R^{C}$. This vector w is then applied to the original features X via element-wise multiplication (denoted by ⊗ in the final step of the diagram) to achieve adaptive feature channel recalibration:
  (4) $$X_{out}=X⊗w$$
  where $X_{out}$ is the final output feature map, X is the input feature map from the C2f module, w is the computed channel attention vector, and ⊗ denotes element-wise multiplication.

Integrating MCAttn into C2f modules fundamentally aims to significantly improve the quality of the network’s feature representations. We anticipate that this enhancement will: (1) strengthen the model’s ability to discriminate features of defect targets in GIS X-ray images that vary widely in size, morphology, and signal-to-noise ratio; (2) improve the model’s generalization performance and robustness to interferences such as noise by introducing randomness during training; and (3) more effectively suppress task-irrelevant background features, enabling subsequent network layers to focus on information more pertinent to defect classification and localization.

### 2.3. PPA Module

At deeper backbone stages (e.g., the P5 stage, Layer 8 in Figure 1), repeated downsampling causes feature maps to progressively lose high-frequency spatial details essential for identifying minute defects, even as they gain semantic richness. Standard C2f modules often fail to balance the aggregation of this semantic context with the preservation of fine-grained features. To overcome this limitation, this paper replaces the C2f module at this stage with the Parallelized Patch-Aware Attention (PPA) module [23]. Originally designed for infrared small target detection, PPA utilizes a parallel multi-branch architecture to capture features at multiple scales, which are then refined using subsequent attention mechanisms.

**Figure 3:**
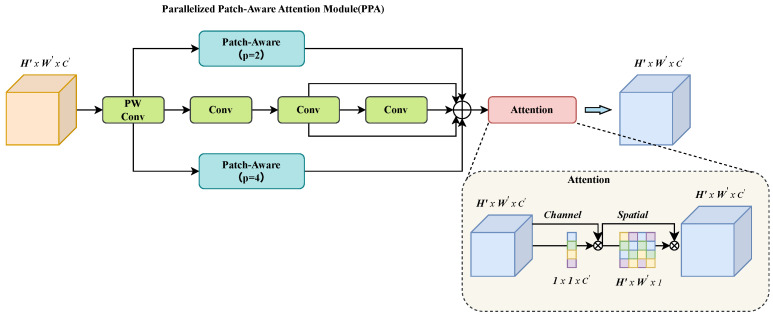
Structure of PPA module.

The overall structural design of the PPA module is illustrated in Figure 3 and primarily consists of two core stages:

(1) Parallel Multi-Branch Feature Extraction: For an input feature F′ (typically obtained after channel adjustment via an initial 1 × 1 convolution), PPA employs three parallel branches to capture information at different levels and scales:

- Local Perception Branch (Patch-Aware, p=2): This branch divides the input feature map into 2×2 local image patches and independently learns and interacts with the embedding representation of each patch (e.g., through a Feed-Forward Network (FFN) and task-correlation-based feature selection mechanisms [23,25]). It ultimately reconstructs a feature map $F_{local}$ that focuses on local details.
- Global Perception Branch (Patch-Aware, p=4): Employing a similar mechanism but with larger 4×4 image patches, this branch aims to capture broader contextual dependencies, outputting a feature map $F_{global}$. This patch-aware processing allows the model to perform adaptive feature aggregation across different spatial extents.
- Serial Convolution Branch: This branch serves to capture classic local features with strong translation invariance, complementing the patch-based processing of the other two branches. It consists of a stack of several standard 3×3 convolutional layers, each typically followed by Batch Normalization (BN) and a ReLU activation function. This design ensures the extraction of robust, low-level feature patterns such as edges and textures, which are then fused with the multi-scale contextual information from the parallel branches to form a more comprehensive feature representation, yielding $F_{conv}$.

The selection of patch sizes p=2 and p=4 is a deliberate design choice to establish a multi-scale perception mechanism. The smaller 2×2 patches enable the model to focus on fine-grained local features, which is critical for identifying the subtle textures and edges of small defects. Conversely, the larger 4×4 patches provide a broader receptive field, allowing the model to capture more extensive contextual information and better distinguish defects from the complex surrounding background structures. By processing these two scales in parallel, the PPA module can simultaneously perceive both detailed and contextual information, which is highly beneficial for the GIS defect-detection task. The outputs of these three branches are subsequently fused to obtain the aggregated feature $\tilde{F}=F_{local}+F_{global}+F_{conv}$.

(2) Attention-based Feature Enhancement: As illustrated in the latter stage of Figure 3, the aggregated feature $\tilde{F}$ is fed into a cascaded attention module for further refinement. This two-stage process is designed to first determine “what” features are important, and then identify “where” they are located.

- Channel Attention: First, the features pass through an Efficient Channel Attention (ECA-Net) [26] module. ECA-Net adaptively recalibrates the importance of each channel by learning cross-channel interaction without dimensionality reduction, effectively highlighting which feature channels are most relevant to the defect-detection task. This yields a channel-refined feature map $F_{c}=M_{c}\left(\tilde{F}\right)⊗\tilde{F}$, where $M_{c}$ is the channel attention map.
- Spatial Attention: Subsequently, the channel-refined feature map $F_{c}$ is processed by a spatial attention mechanism, similar to the one used in CBAM [27]. This module generates a 2D spatial attention map that emphasizes the most informative regions within the feature map, guiding the model to focus on the precise locations of potential defects while suppressing background noise. The final output is produced by $F_{s}=M_{s}\left(F_{c}\right)⊗F_{c}$, where $M_{s}$ is the spatial attention map.

Here, ⊗ denotes element-wise multiplication. This cascaded channel-then-spatial attention structure ensures a comprehensive feature refinement, equipping the backbone network with stronger and more robust feature extraction capabilities for subsequent detection stages.

In the context of the GIS X-ray defect-detection task in this paper, replacing the C2f module in the deeper layers of the backbone with the PPA module offers several key advantages. (1) The multi-branch parallel processing can effectively capture both local detail features of defects (such as edges and textures) and their global background structural information simultaneously. (2) The Patch-Aware mechanism provides the model with the ability to learn and interact with features at different spatial granularities, which is beneficial for handling defects of varying sizes. (3) The subsequent dual channel and spatial attention mechanisms can further refine features, amplifying defect-related signals while suppressing irrelevant information common in X-ray images, such as noise or artifacts. Therefore, the introduction of PPA is expected to equip the backbone network with stronger and more robust feature extraction capabilities, thereby improving performance in subsequent detection stages.

### 2.4. Enhanced Feature Pyramid Neck

**Figure 4:**
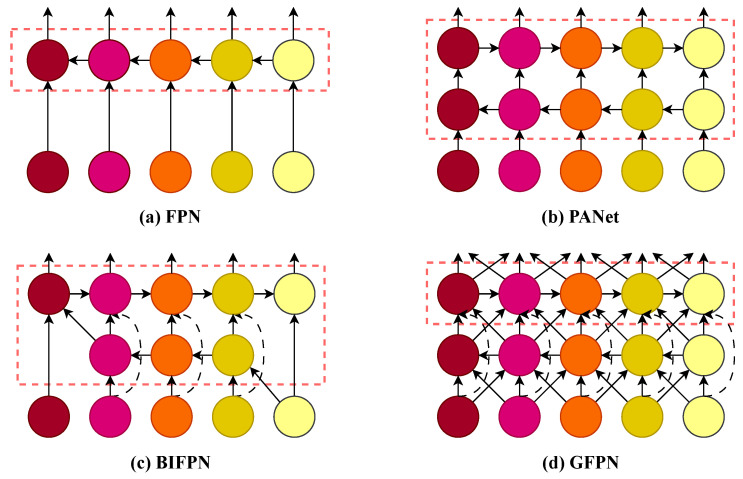
Evolution of feature pyramid networks.

In object detection, effectively fusing features from different backbone levels is crucial for identifying targets across various scales. Over the years, several feature pyramid network architectures have been proposed to address this challenge, with their evolution illustrated in Figure 4. The classic Feature Pyramid Network (FPN) [20] (Figure 4a) established a top-down pathway to merge high-level semantic features with low-level spatial details. Subsequently, the Path Aggregation Network (PANet) [13] (Figure 4b) introduced an additional bottom-up pathway, creating a more effective bidirectional information flow. More advanced structures like BiFPN [28] (Figure 4c) and GFPN [21] (Figure 4d) further optimized the fusion process.

Our proposed neck network is designed by drawing upon the strengths of these established architectures. In terms of path topology, we adopt the proven bidirectional architecture of PANet (as shown in Figure 4b) to ensure robust two-way fusion of semantic and spatial information. However, our core design philosophy is inspired by the “heavy-neck” paradigm emphasized in GFPN (Figure 4d). The central idea of GFPN is that investing more computational resources and complexity in the neck network’s fusion nodes leads to more powerful and discriminative multi-scale feature representations.

In line with this philosophy, instead of using simple convolutional layers for fusion, our neck employs more powerful CSPStage modules within both the top-down and bottom-up pathways (as detailed in Figure 1). This use of complex feature processing units at each fusion stage is our key implementation of the “heavy-neck” concept. The specific pathways are as follows:

- Top-down Pathway: Deep, high-level semantic features are progressively upsampled and fused with shallower features from the backbone. Each fused feature map is then processed by a CSPStage module to refine the representation.
- Bottom-up Pathway: Subsequently, the refined, semantically-rich feature maps from the top-down path are progressively downsampled and fused with features from higher levels. These are also processed by CSPStage modules to enrich deeper feature maps with precise localization cues.

By combining a PANet-like bidirectional path structure with the GFPN-inspired “heavy-neck” design, our enhanced neck network achieves a more thorough and effective fusion of multi-scale features, significantly improving the model’s performance on detecting GIS defects.

## 3. Experiments and Result

### 3.1. Dataset

All experiments in this paper were conducted on a custom-built X-ray image dataset for internal defects in Gas-Insulated Switchgear (GIS) equipment, provided by the State Grid Ningxia Electric Power Co., Ltd. (Yinchuan, Ningxia, China). This dataset, reflecting real-world industrial application scenarios, comprises a total of 718 X-ray images covering various equipment models and imaging conditions.

To support supervised learning, all images were annotated by professional engineers using bounding boxes. This study focuses on five representative types of internal GIS defects. A detailed description of their visual characteristics, along with the distribution of annotated instances for each category, is presented in Table 1. The dataset exhibits a natural class imbalance, which is typical of real-world industrial data where some defect types occur more frequently than others.

**Figure 5:**
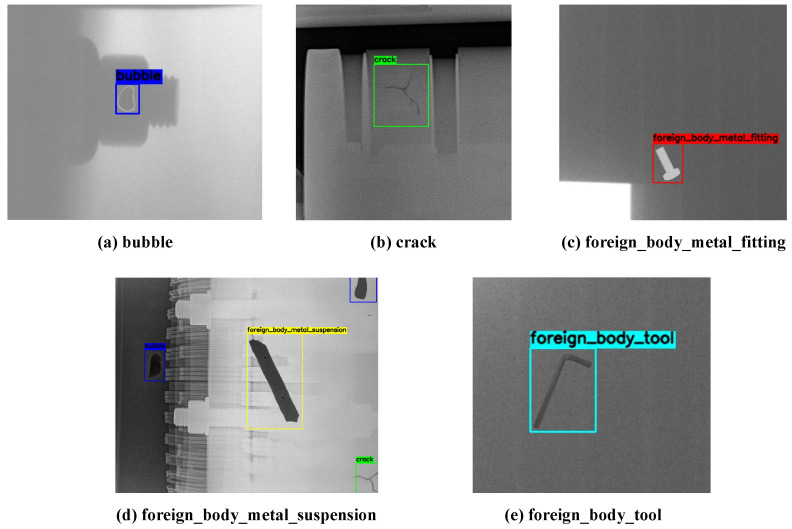
Example images from the custom GIS X-ray dataset, showcasing different defect types discussed in Table 1.

The collected 718 annotated images were randomly partitioned into a Training Set, a Validation Set, and a Test Set according to an 8:1:1 ratio. Example images illustrating each of these defect types are shown in Figure 5.

### 3.2. Experimental Environment

All models in this paper were trained under the PyTorch 2.3.0 framework. Stochastic Gradient Descent (SGD) was employed as the optimizer, with an initial learning rate set to 0.01 and a batch size of 32. All models were uniformly trained for 300 epochs. For other training hyperparameters, the default configuration of YOLOv10n was adopted. The specific hardware and software environment configurations for the experiments are detailed in Table 2.

Recognizing that overfitting is a primary challenge when training on specialized datasets of limited size, we implemented a multi-layered strategy to ensure the model’s generalization ability. This strategy consisted of three key components:

- Transfer Learning: All models were initialized with weights pre-trained on the large-scale COCO dataset. This provides the network with a robust foundation of general visual features, preventing it from having to learn these from scratch and significantly reducing the risk of fitting to noise in our specific dataset.
- Extensive Data Augmentation: We utilized a rich set of online data augmentation techniques, including mosaic augmentation, random affine transformations (rotation, scaling, translation), and color space adjustments (HSV). This effectively creates a larger and more diverse “virtual” dataset, forcing the model to learn invariant and robust features rather than memorizing the training examples.
- Regularization: A standard weight decay of 0.0005 was applied during optimization. This technique penalizes large weights in the network, thereby constraining model complexity and discouraging it from learning overly complex patterns that are specific only to the training data.

The effectiveness of this comprehensive strategy is ultimately validated by the strong performance our model achieved on the unseen, held-out test set. A model that had significantly overfit would fail to generalize and perform well on this data; thus, the high accuracy reported in our results serves as compelling empirical evidence that overfitting was successfully mitigated.

The inference speed, measured in Frames Per Second (FPS), was evaluated on the same NVIDIA GeForce RTX 4090 GPU with a batch size of 1 to reflect real-world deployment performance.

### 3.3. Experimental Metrics

To comprehensively and quantitatively evaluate the performance of the proposed improved model and other comparative methods on the GIS X-ray defect-detection task, we adopted standard evaluation metrics recognized in the field of object detection. The calculation of these metrics is typically based on the following four fundamental statistics: True Positives (TP), False Positives (FP), False Negatives (FN), and True Negatives (TN) (though TN is less directly used in object detection). In this task:

- TP: Correctly detected defects (the IoU between the predicted bounding box and the ground truth bounding box is greater than a set threshold τ, and the predicted class matches the true class).
- FP: Incorrect detection results (the IoU between the predicted box and all ground truth boxes is less than the threshold τ, or the predicted class is incorrect, or background is misidentified as a defect).
- FN: Undetected true defects (a ground truth defect box exists, but no predicted box matches it with a sufficiently high IoU (≥τ) and the correct class).

IoU is a key metric for measuring the degree of overlap between a predicted bounding box $B_{p}$ and a ground truth bounding box $B_{gt}$. Its calculation formula is:

(5) $$\mathrm{IoU}\left(B_{p},B_{gt}\right)=\frac{\mathrm{Area}(B_{p}\cap B_{gt})}{\mathrm{Area}(B_{p}\cup B_{gt})}$$

The value range of IoU is [0, 1], where a higher value indicates better overlap between the predicted and ground truth boxes.

Based on the above fundamental definitions, we selected the following core metrics for model evaluation:

- **Precision (P):** The proportion of actual true defects among all samples predicted as defects by the model. It measures the accuracy of the model’s predictions.
  (6) $$P=\frac{\mathrm{TP}}{\mathrm{TP}+\mathrm{FP}}$$
- **Recall (R):** The proportion of true defect samples successfully detected by the model among all actual true defect samples. It measures the model’s ability to find all relevant targets (completeness).
  (7) $$R=\frac{\mathrm{TP}}{\mathrm{TP}+\mathrm{FN}}$$
- **Mean Average Precision (mAP):** This is the most important and commonly used comprehensive evaluation metric in object-detection tasks. For a single class *i*, its Average Precision (${\mathrm{AP}}_{i}$) is typically defined as the average of precision values at different recall levels, which can be obtained by calculating the area under the Precision–Recall (PR) curve. The mAP is then the arithmetic mean of the AP values for all $N_{c}$ classes:
  (8) $$\mathrm{mAP}=\frac{1}{N_{c}}\sum_{i=1}^{N_{c}}{\mathrm{AP}}_{i}$$
  Since the calculation of AP depends on the choice of the IoU threshold τ, we follow the convention of international standard competitions and primarily report the following two mAP metrics:
  – **mAP@0.5 (or mAP50):** The mAP calculated at a single IoU threshold τ=0.5. This is a widely adopted standard in the PASCAL VOC challenge [29].
  – **mAP@0.5:0.95 (or mAP[0.5:0.95]):** The mAP calculated by taking multiple IoU thresholds τ from 0.5 to 0.95 in steps of 0.05 (i.e., 0.5, 0.55,…, 0.95), calculating mAP for each threshold, and then averaging these mAP values. This is the primary evaluation metric used in the COCO dataset challenge [30], and it imposes higher requirements on the localization accuracy of targets.

Through a comprehensive analysis of these metrics, we can thoroughly assess the model’s accuracy, recall capability, and localization precision in the GIS defect-detection task.

### 3.4. Ablation Experiments and Results

To systematically validate our design choices and quantify the contributions of the proposed components, we conducted two sets of ablation studies on the custom-built GIS-Xray dataset.

First, we performed a progressive ablation study starting from the lightweight YOLOv10n baseline. These studies involved incrementally introducing and combining our four key enhancements: (1) the NWD loss function; (2) the MCAttn mechanism; (3) the PPA module; and (4) the GFPN-inspired neck. All experiments were conducted under identical training configurations. The detailed results are summarized in Table 3.

The experimental results in Table 3 clearly demonstrate the positive impact of each improved component on model performance. First, comparing Experiment 2 with Experiment 1 (BASE), the standalone introduction of the NWD loss function, without increasing any parameter count or computational load, improved mAP@0.5 by 3.5% and mAP@0.5:0.95 by 3.2%. This fully validates the significant advantage of the NWD loss function in improving the localization of minute defect targets. Second, examining the backbone enhancements, the C2fMCAttn module (Experiment 3) and the PPA module (Experiment 4) both improved performance, with PPA showing slightly higher gains at the cost of more parameters. Next, the GFPN-inspired neck network (Experiment 5) increased mAP@0.5:0.95 by 3.7%, proving the effectiveness of the improved fusion strategy. Finally, after progressively combining the components (Experiments 6, 7, and 8), the model’s performance continuously improved, with our final model achieving a 5.0% improvement in mAP@0.5:0.95 over the baseline.

Furthermore, to validate the superiority of our chosen modules over other mainstream alternatives, we conducted a second set of comparative experiments, with the results presented in Table 4. The data show that our C2f_MCAttn module achieves a higher mAP@0.5:0.95 score (0.653) compared to both SE (0.631) and CBAM (0.635). Similarly, our GFPN (0.661) outperforms the popular BiFPN structure (0.654). These comparisons provide strong justification for our specific design choices.

In summary, the results from both sets of ablation experiments strongly demonstrate that the NWD loss function, the backbone network enhancement strategy based on MCAttn and PPA, and the GFPN-inspired neck network structure proposed in this paper each contribute significantly to the final performance. Not only are they effective when combined synergistically, but they also prove to be superior choices compared to other common alternatives for the GIS X-ray defect-detection task. Of course, the performance improvement is accompanied by a moderate increase in model complexity, reflecting a potential trade-off between accuracy and efficiency in practical applications.

### 3.5. Model Comparison and Visualization Analysis

To comprehensively evaluate the proposed model, we conducted both an in-depth per-category analysis against the baseline and a broad performance comparison against other mainstream lightweight models.

First, to delve deeper into the specific contributions of our improvements, we analyzed the per-category performance gains of our final model over the baseline, as detailed in Table 5. The results show that our model achieves consistent improvements across all defect categories. Most notably, a remarkable gain of **9.7 percentage points** in AP is observed for the “Crack” class. This is a particularly important finding, as cracks are often characterized by their slender, elongated shapes and low contrast, making them one of the most challenging defect types to detect. This substantial improvement strongly suggests that our key enhancements—such as the NWD loss function tailored for small and slender objects, and the advanced attention mechanisms (MCAttn and PPA) for superior feature extraction—are highly effective in addressing the core difficulties of this detection task.

Next, to further validate the effectiveness and advancement of our complete model, we conducted a comprehensive performance comparison against several other mainstream lightweight real-time object-detection models on the GIS-Xray test set. The models included in the comparison were YOLOv3-tiny [9], YOLOv5n [11], YOLOv8n [14], and YOLOv9s [15]. The main quantitative evaluation results are summarized in Table 6.

The comparison results in Table 6 clearly show that the model proposed in this paper significantly surpasses the baseline and other compared models in accuracy. Specifically, compared to the direct baseline YOLOv10n, our model achieved a 4.6% improvement in mAP@0.5 and a 5.0% improvement in mAP@0.5:0.95. This significant accuracy gain was realized with a moderate increase in model complexity (from 8.2 to 10.2 GFLOPs) and a corresponding reduction in inference speed (from 103 to 85 FPS). This demonstrates an excellent trade-off between accuracy and real-time performance, as our model maintains a high inference speed well-suited for practical applications.

The advantages of our method are more pronounced when compared with other representative models. For instance, while YOLOv8n and YOLOv10n offer higher speeds, our model provides a substantial lead in accuracy (e.g., +7.3% mAP@0.5:0.95 over YOLOv8n), making it a more compelling choice for high-precision applications. Compared to bulkier models like YOLOv3-tiny and YOLOv9s, our model is not only significantly more accurate but also more than twice as fast, further highlighting the efficiency of our design. Overall, for the studied GIS X-ray defect-detection task, our model achieves an optimal balance between detection accuracy and inference speed.

**Figure 6:**
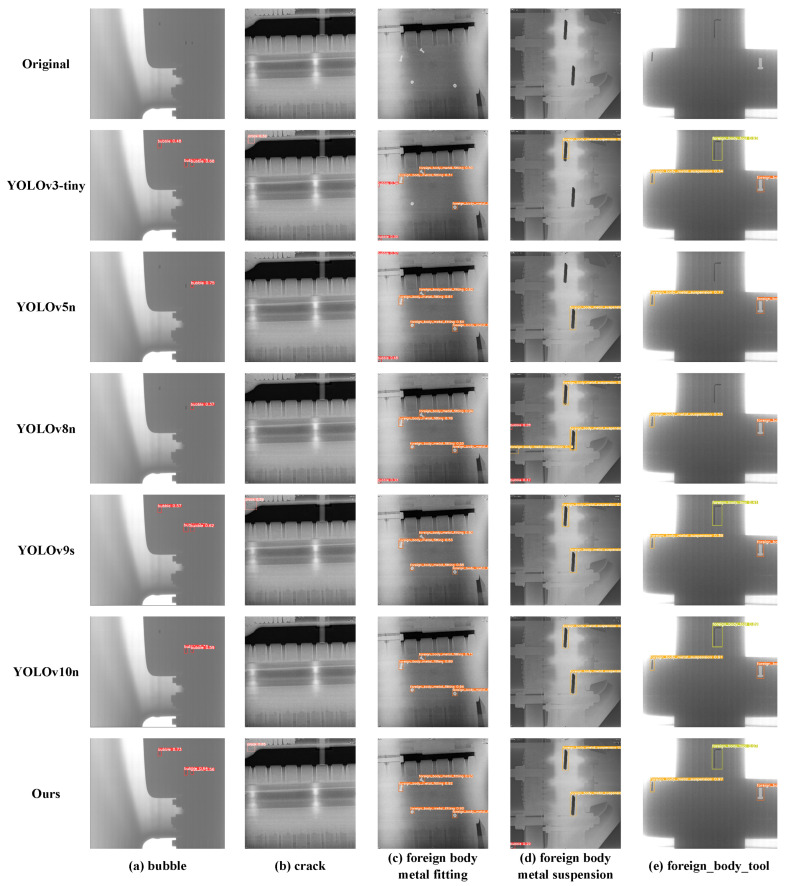
Comparison of different models.

In addition to the quantitative performance metrics evaluated above, to more intuitively compare the performance differences of various models in actual detection scenarios, we selected typical GIS X-ray image samples covering all five defect categories and conducted a visual analysis of their detection results. Figure 6 shows a comparison of the specific detection outputs of our proposed model (Ours) against the baseline YOLOv10n and four other advanced lightweight detectors (YOLOv3-tiny, YOLOv5n, YOLOv8n, YOLOv9s) on these samples.

Through careful observation and comparison of the detection results from each model in Figure 6, the significant advantages of our proposed model in handling various complex situations can be clearly seen:

For the minute bubble defect in Figure 6a, its features are faint, making detection extremely difficult. Interestingly, YOLOv3-tiny, with its larger parameter count, can detect this defect relatively well, whereas newer models like YOLOv5n and YOLOv8n exhibit missed detections. This suggests that YOLOv3-tiny performs adequately in handling certain small defects. In contrast, our proposed model (Ours) not only stably detects this minute bubble but also does so with high prediction confidence (as indicated by the bounding box). This visually demonstrates that our model’s performance in handling such challenging minute, low-contrast defects is significantly superior to most other lightweight comparative models.

The model’s superiority in detecting challenging defects is particularly evident with the concealed, hairline crack in Figure 6b. Due to its extremely subtle features, most competing models failed to achieve a successful detection. It is noteworthy that, apart from our proposed model, only the significantly bulkier YOLOv3-tiny (10.3 M Params) and YOLOv9s (7.3 M Params) managed to identify this feature. This result highlights our architecture’s exceptional accuracy-efficiency trade-off, proving its ability to outperform larger models on such critical, fine-grained defects while maintaining a lightweight profile.

When detecting “foreign body (metal fitting)” type targets (Figure 6c), most models performed successfully. However, misdetections are a concern; for instance, YOLOv8n incorrectly identified background as a “bubble” in this sample. Similarly, in the detection of “foreign body (metal suspension)” (Figure 6d), other models also showed potential misdetections. It must be objectively acknowledged that while our model generally performs stably, it may occasionally produce a few False Positives in certain situations (as might be observed in Figure 6d), indicating that there is still room for further optimization in suppressing misdetections.

Finally, for the “foreign body (tool)” defect in Figure 6e, which is larger and has more distinct features, all compared models performed well, accurately detecting and localizing it, as expected.

Overall, these instances clearly demonstrate that through synergistic optimization of the loss function, backbone network attention mechanisms, and neck feature fusion structure, our model can more effectively cope with various complex situations in GIS X-ray images. It particularly exhibits significant advantages in detecting minute, low-contrast, and densely packed defects, thereby validating the effectiveness and advancement of the proposed method.

## 4. Conclusions

This research tackles the critical challenge of detecting micro-defects in GIS X-ray images, which are inherently characterized by submillimeter scales, low contrast, and cluttered backgrounds. The aim was to propose an automated detection method with higher precision and robustness.

To this end, we proposed a significantly improved model based on the YOLOv10n framework. This model enhances detection performance through multifaceted synergistic optimizations: (1) adopting the NWD loss function effectively improved the localization capability for small targets; (2) embedding MCAttn into the C2f modules of the backbone and introducing PPA to replace deeper modules significantly enhanced the extraction and discrimination of key features; and (3) leveraging the design principles of GFPN, a new neck network structure was built, optimizing the efficiency and effectiveness of multi-scale feature fusion.

A series of experiments on a real-world GIS X-ray image dataset, including detailed ablation studies and comparative analyses with several advanced lightweight detectors (such as YOLOv3-tiny, YOLOv5n, YOLOv8n, and YOLOv9s), validated the effectiveness of our method. The results indicated that our complete proposed model significantly outperformed the baseline YOLOv10n and other comparative models across various evaluation metrics. For instance, on the mAP@0.5:0.95 metric, it achieved a 5.0% improvement over YOLOv10n, reaching a state-of-the-art level of 0.674. Qualitative analysis also intuitively demonstrated our model’s advantages in detecting challenging samples, especially minute and slender defects.

The proposed improvements and experimental results from this research demonstrate that by targeted optimization of the loss function, introduction of advanced attention mechanisms, and enhancement of the feature fusion structure, the performance of object-detection models in specific industrial X-ray imaging scenarios can be effectively boosted. This work holds significant theoretical reference value and potential application prospects for advancing intelligent GIS condition assessment technology, improving power grid operational and maintenance efficiency, and ensuring the safe and reliable operation of power systems.

Concurrently, we recognize that while our method brings accuracy improvements, it also leads to an increase in model parameter count and computational complexity. Furthermore, occasional misdetections in certain complex backgrounds suggest that there is still room for improvement in the model’s feature discrimination capabilities and background suppression strategies.

Future research will aim to build upon and broaden the findings of this study. Key directions include: (1) validating and further improving the model on larger and more diverse GIS X-ray datasets as they become available through our ongoing industrial collaboration, which will further enhance the model’s robustness and generalization ability within its primary application domain; (2) comparing and adapting our proposed enhancements to newer architectures like YOLOv11 and YOLOv12 to stay at the forefront of the field; (3) further exploring model lightweighting techniques and researching strategies to reduce the false alarm rate, thereby enhancing the model’s practical deployment value.

## Figures and Tables

**Table 1:** Description and distribution of defect categories in the dataset.

Defect Category	Visual Characteristics in X-Ray Images	Instances
Bubble	Small, localized dark region (low density) with a defined shape, representing a gas void.	215
Crack	Fine, irregular dark line (low density) with very low contrast, representing a fracture.	189
Foreign body metal fitting	High-density (bright white) object with a regular geometric shape (e.g., screw).	452
Foreign body metal suspension	Slender or rod-like dark object (low density), often appearing near complex structures like insulators.	378
Foreign body tool	Large, high-density object with a recognizable tool-like shape.	281
Total		1515

**Table 2:** Hardware environment and software configuration.

Environment	Configuration
Hardware	
CPU	Intel^®^ Core™ i7-13700KF
GPU	NVIDIA^®^ GeForce^®^ RTX 4090 (24 GB GDDR6X)
RAM	64 GB DDR5
Software	
Operating System	Windows 11
CUDA	12.4
cuDNN	9.8.0
Pytorch	2.3.0
Python	3.11

**Table 3:** Results of ablation experiments.

Experiment	Model	P	R	mAP@0.5	mAP@0.5:0.95	Params (M)	GFLOPs
1	YOLOv10n (BASE)	0.832	0.837	0.904	0.624	2.70	8.2
2	BASE + NWD	0.883	0.904	0.939	0.656	2.70	8.2
3	BASE + C2fMCAttn	0.902	0.850	0.932	0.653	2.76	8.4
4	BASE + PPA	0.911	0.903	0.948	0.658	4.41	9.5
5	BASE + GFPN	0.910	0.921	0.937	0.661	3.32	8.8
6	BASE + NWD + C2fMCAttn	0.920	0.875	0.930	0.650	2.76	8.4
7	BASE + NWD + C2fMCAttn + PPA	0.924	0.895	0.947	0.668	4.48	9.7
**8**	**Ours**	**0.949**	**0.912**	**0.950**	**0.674**	**5.10**	**10.2**

**Table 4:** Comparison of the proposed modules with other mainstream modules.

Model Configuration	P	R	mAP@0.5	mAP@0.5:0.95
***Attention Module Comparison on Backbone***				
YOLOv10n (Baseline) + SE	0.875	0.832	0.915	0.631
YOLOv10n (Baseline) + CBAM	0.889	0.841	0.919	0.635
YOLOv10n (Baseline) + C2f_MCAttn	**0.902**	**0.850**	**0.932**	**0.653**
***Neck Structure Comparison***				
YOLOv10n (Baseline) + BiFPN	0.892	0.901	0.911	0.654
YOLOv10n (Baseline) + GFPN	**0.910**	**0.921**	**0.937**	**0.661**

**Table 5:** Per-category Average Precision (AP@.5) comparison between the baseline model and our final proposed model.

Defect Category	YOLOv10n (Baseline)	Ours	Improvement
Bubble	0.898	0.935	+0.037
Crack	0.780	0.877	**+0.097**
Foreign body metal fitting	0.960	0.994	+0.034
Foreign body metal suspension	0.941	0.978	+0.037
Foreign body tool	0.938	0.967	+0.029
**mAP@0.5**	**0.904**	**0.950**	**+0.046**

**Table 6:** Model performance and speed comparison of YOLO series.

Model	P	R	mAP@0.5	mAP@0.5:0.95	Params (M)	GFLOPs	FPS (Hz)
YOLOv3-tiny	0.889	0.843	0.854	0.611	10.3	31.5	40
YOLOv5n	0.801	0.777	0.811	0.597	2.6	18.9	60
YOLOv8n	0.841	0.787	0.823	0.601	3.1	8.1	105
YOLOv9s	0.876	0.812	0.883	0.632	7.3	27.6	45
YOLOv10n	0.832	0.837	0.904	0.624	2.70	8.2	103
**Ours**	**0.949**	**0.912**	**0.950**	**0.674**	**5.10**	**10.2**	**85**

## Data Availability

The raw X-ray image dataset used in this study is not publicly available due to business confidentiality and proprietary restrictions from our industrial partner, State Grid Ningxia Electric Power Co., Ltd. However, the complete source code, model configurations, and the final trained model weights are publicly available on GitHub at https://github.com/imxiaolong0804/YoloV10Improve (accessed on 24 August 2025).
